# A Comparison of the Specific Facial Trauma Cases at the Department of Maxillofacial Surgery, Gdansk, Poland, from March 2019 to August 2023

**DOI:** 10.3390/jcm13113095

**Published:** 2024-05-25

**Authors:** Marta Bień, Barbara Drogoszewska, Adam Polcyn, Adam Michcik, Łukasz Garbacewicz

**Affiliations:** Department of Maxillofacial Surgery, Medical University of Gdansk, Mariana Smoluchowskiego 17, 80-214 Gdansk, Poland; drog@gumed.edu.pl (B.D.); adampolcyn@gumed.edu.pl (A.P.); adammichcik@gumed.edu.pl (A.M.); lgarbacewicz@gumed.edu.pl (Ł.G.)

**Keywords:** COVID-19, maxillofacial trauma, location and frequency of trauma

## Abstract

**Background:** Accidents involving the maxillofacial area are sudden and unforeseen, such as traffic accidents and physical altercations. The COVID-19 pandemic was a critical threat to the public in aspects not only involving physical health but also those affecting psychological health due to isolation, leading to a higher incidence of stress and depression among the general population and specifically in patients with OMF trauma. This study assessed the relationship between the impact of the COVID-19 pandemic and the quantity and severity of maxillofacial injuries. **Methods**: Data were retrieved from the Department of Maxillofacial Surgery of the University Clinical Centre in Gdansk from March 2019 to August 2023. **Results:** There was an increased risk of injury occurrence to the condylar process of the mandible, especially the left side, Le Fort type II/III fractures, injuries of the maxillary alveolar process, and displacement of the upper facial mass. Simultaneously, a decreased occurrence of certain injuries i.e., Le Fort type III fractures and Le Fort type I/II fractures, was recorded. **Conclusions**: The COVID-19 pandemic led to an increased occurrence of high-energy injuries, including displacement of the upper facial mass (*p* = 0.010).

## 1. Introduction

Coronaviruses affect both humans and animals, causing mild to severe upper respiratory tract illnesses [1]. In Wuhan, China, a severe respiratory syndrome known as Coronavirus 2 (SARS-CoV-2) was reported in December 2019, which led to the coronavirus outbreak that spread to many countries across the globe [2].

An analysis of the developmental dynamics of the SARS-CoV-2 pandemic indicated that the beginning of the epidemic occurred later in Poland than in other countries of the European Union, and generally at the same time regionally. The reasons for this included the geographical and economic location, as well as the nature of international passenger transport. Poland is located peripherally to the largest airports within the European Union, which were critical for the very rapid intercontinental spread of the virus. The first confirmed case of the virus in Poland was recorded on 4 March 2020, leading to the announcement of an epidemiological threat by the Minister of Health (2020) between March 14 and March 20, and, finally, the classification of an epidemic as of 20 March 2020. Following these announcements, Polish universities suspended classes on March 10, and by March 12, the universities had closed. Subsequently, access to places capable of hosting the population and public transportation was limited by the regulations of the Minister of Health as of 24 March 2020 [3].

In the context of assessing the impact of the COVID-19 pandemic on various aspects of social life and public health, it is important to understand how these unusual circumstances influenced the conditions for the development of craniofacial injuries. The impact of the pandemic on risk behaviour was complex and multidimensional. These injuries are often the result of accidents, acts of violence, or other traumatic events, and their frequency and severity may be modified by socioeconomic factors and changes in behaviour [4]. During the pandemic, lifestyle changes, restrictions introduced to control the spread of the virus, and, consequently, loneliness, depression, fear of infection, and anxiety secondary to a change in lifestyle could have significantly affected the patterns of risky behaviour [5]. The stress related to the pandemic, social isolation, changes in work organisation, and economic uncertainty (high unemployment rates) may have led to an increase in the consumption of psychoactive substances, which in turn could have increased the risk of injuries [4]. This study involved a male-predominant cohort, with a mean patient age of 34.5 years and an age range of 4–96 years old, allowing for the inclusion of a wide spectrum of the population.

During the COVID-19 pandemic, social restrictions and mass adjustments to public health regulations led to a shift in the patterns of human activity and behaviour. With these adaptations, patterns in maxillofacial trauma were consequently interrupted [6]. There was a lack of studies in the literature assessing the effects of the COVID-19 pandemic on traumatic injuries, and more specifically OMF trauma cases, necessitating research allowing for a better understanding of how epidemics affect the severity and frequency of facial traumas.

The purpose of this article was to assess the impact of coronavirus infections (SARS-CoV-2) in the period from March 2020 to May 2022 on the quantity and severity of facial cranial injuries in patients treated at the Department of Maxillofacial Surgery.

This comparison of the quantity and severity of injuries to the facial part of the skull of patients treated at the Department of Maxillofacial Surgery during the period of the highest dynamics of coronavirus (SARS-CoV-2) infections was completed by considering the period from March 2019 to February 2020 and the period from June 2022 to August 2023.

## 2. Materials and Methods

A retrospective analysis was conducted on non-randomised data from 704 patients with facial trauma who were hospitalised in the Department of Maxillofacial Surgery of the University Clinical Centre in Gdansk. Research materials were obtained from archived medical documentation. Sensitive personal data were removed and the rules of the GDPR and Personal Data Protection Policy at MUG were followed. The patients were not given financial compensation to be a part of this study. Consent was obtained from the Director of the University Clinical Centre and the Bioethics Committee. The methodology, formal analysis, and observations were made by one clinician, Dr Marta Bień. 

Data were collected between March 2019 and August 2023. The data were divided into two distinct time frames for analysis during different phases of the COVID-19 pandemic:

- The period of peak pandemic intensity: 03/2020 to 05/2022, covering the emergence of infections and their constant increase. The cohort consisted of 247 patients, representing 35.4% of the total sample.
- The pre- and post-pandemic period: 03/2019 to 02/2020 and 06/2022 to 08/2023, respectively, with an eventual return to a sense of stability. The number of patients was 457, corresponding to 64.6% of the total study sample.

The collected data were statistically analysed using Fisher’s exact test, Pearson’s chi-squared test, and the proportion test. The number (*n*) and percentage of individual categories were also assessed. Results at *p* < 0.05 were considered significant.

Analysis was conducted using the R statistical language (version 4.3.1; R Core Team, 2023).

## 3. Results

The distribution of sociodemographic parameters in the study cohort was as follows:

- In the analysis of gender distribution, in both periods, a statistically insignificant difference was observed (*p* = 0.151). During the pandemic, men constituted 77.33% of the sample (191), while outside the pandemic, this percentage increased to 81.84% (374). Women constituted 22.67% (56), and in the non-pandemic period, this percentage decreased to 18.16% (83).
- The statistical analysis of the study sample in total and divided into periods related to the COVID-19 pandemic did not show statistically significant differences in the age of the patients (*p* = 0.782). The median age of patients during the pandemic was 32 years (1st quartile 24, 3rd quartile 48), while outside the period of the pandemic, the median was 33 years (1st quartile 24, 3rd quartile 44), suggesting a slight change in median age that was not statistically significant.

The table presented below lists the characteristics of the injuries seen during the pandemic, including the location. 

Fractures within the mandible can be divided into the following:

- Fracture of the angle of the mandible (left side, bilaterally, right side);
- Fracture of the body of the mandible in the middle;
- Mandibular body fracture (left side, bilaterally, right side);
- Fracture of the mandibular ramus;
- Fracture of the condylar process of the mandible (left side, bilaterally, right side);
- Fracture of the head of the mandible;
- Fracture of the coronoid process of the mandible;
- Fracture of the alveolar part of the mandible.

Jaw fractures:

- Le Fort type I;
- Le Fort type II;
- Le Fort type III;
- Le Fort type I/II;
- Le Fort type II/III;
- Fracture of the maxillary alveolar process;
- Fracture in the maxillary sinus;
- Displacement of the upper facial mass.

Other:

- Zygomaticomaxillary orbital fracture;
- Fracture of the zygomatic arch;
- Fracture in the orbit;
- Fracture of the nasal bone;
- Fracture in the frontal bone;
- Multi-site head and facial fractures.

The data are shown in Table 1.

Injuries of the angle of the mandible were identified in 195 patients. The most frequently reported were injuries to the left side of the mandible, which accounted for 61.54% of cases in the total sample. The comparative analysis showed slight differences in the percentage of injuries to the left side of the mandible between the pandemic period (62.90%) and the non-pandemic period (60.90%), but this difference was not statistically significant (*p* = 0.623). Bilateral injuries were much less common, accounting for 4.10% of the overall sample, with 1.61% of cases occurring during the pandemic and 5.26% outside of it. In the case of injuries to the right side of the mandible, the percentage was 34.36% in the entire sample, with slight fluctuations between the pandemic period (35.48%) and the non-pandemic period (33.83%).

Medial mandibular body injuries were recorded in the entire sample of 704 patients. Most (90.06%) did not show any medial damage to the mandibular body. The comparison between the pandemic and non-pandemic periods also showed no significant statistical differences (*p* = 0.907), with corresponding values of 89.88% during the pandemic and 90.15% outside of it. Injuries with damage to the mandibular body in the middle constituted 9.94% of the total sample, with a slight predominance during the pandemic (10.12%) compared to the non-pandemic period (9.85%).

For injuries of the mandibular body depending on the side affected, 42.86% of cases concerned the left side, 5.04% were bilateral, and 52.10% concerned the right side. The differences between the pandemic and non-pandemic periods were minimal and statistically insignificant (*p* = 0.808), with left-side injury rates of 40.70% during the pandemic and 44.08% in the non-pandemic period, 5.81% bilateral vs. 4.61%, and 53.49% of the right side vs. 51.32%, respectively.

Fractures of the mandibular branch made up 6.11% of the injuries in the total sample, with a proportion of 6.88% during the pandemic decreasing to 5.69% after the pandemic (*p* = 0.528), which was not statistically significant.

Of 164 patients presenting injuries of the condylar process, there was a significant statistical difference observed during the pandemic as opposed to the pre- and post-pandemic periods. During the pandemic, 55.88% of these injuries affected the left side, which was a significant increase when compared to the results of the non-pandemic period of 23.96% (*p* < 0.001). Bilateral injuries of the condylar process occurred less frequently during the pandemic (7.35%) than in the non-pandemic period (58.33%), to a significant degree (*p* < 0.001). For unilateral injuries of the condylar process affecting the right side, during the pandemic, 36.76% of the total sample was seen, in comparison to 17.71% in the non-pandemic period, which was statistically significant (*p* < 0.001).

Mandibular head fractures made up 2.41% of injuries within the total sample, with a frequency of 2.83% during the pandemic, decreasing to 2.19% in the non-pandemic period (*p* = 0.594).

Fractures of the coronoid process of the mandible occurred in 2.41%, with a slightly increased frequency of 3.24% during the pandemic in comparison to 1.97% in the non-pandemic period (*p* = 0.295).

Fractures of the alveolar parts of the mandible were rare and showed no difference in incidence during the pandemic (1.21%) and in the non-pandemic period (1.09%) (*p* = 1.000).

In analysing jaw injuries according to the Le Fort classification, 69 cases were recorded. Le Fort I type fractures accounted for 23.19% of the total number of injuries, with slight differences between the pandemic period (27.27%) and the non-pandemic period (21.28%), which was not statistically significant (*p* = 0.838). In turn, Le Fort II fractures were the most common, accounting for 47.83% of cases. Despite a higher percentage of these fractures occurring during the pandemic (68.18%) compared to the non-pandemic period (38.30%), this difference did not reach statistical significance (*p* = 0.201). However, Le Fort III fractures differed between the analysed periods, with 4.55% of cases occurring during the pandemic, and a significantly higher rate occurring in the non-pandemic period (40.43%), which was statistically significant (*p* = 0.004).

The next segment of data concerns complicated jaw fractures. In this analysis, Le Fort type I/II fractures accounted for 59.02% of cases, with a clear disproportion between the pandemic period (6.25%) and the non-pandemic period (77.78%) (*p* < 0.001). Le Fort type II/III fractures also showed significant differences between both periods, with 93.75% of cases occurring during the pandemic compared to 22.22% in the non-pandemic period.

Injuries to the maxillary alveolar process were reported in 3.41% of the total cases, with a notable increase during the pandemic, where the injury rate was 5.26%, compared to 2.41% outside the pandemic (*p* = 0.046), suggesting a possible influence of factors related to the pandemic increasing the risk of these injuries.

Injuries to the sinus occurred in 4.12% of the studied population, with a frequency of 4.45% during the pandemic and 3.94% during the non-pandemic period (*p* = 0.743), which was not significant.

However, displacement of the upper facial mass, accounting for 2.41% of cases, showed a statistically significant increase during the pandemic (4.45%) compared to the non-pandemic period (1.31%) (*p* = 0.010). This may suggest that pandemic-related circumstances, such as changes in injury patterns resulting from restrictions on professional and social activities, may have contributed to an increased risk of more severe forms of jaw injuries.

For zygomaticomaxillary orbital fractures, which accounted for 18.04% of the injuries, no compelling statistical differences were observed between the pandemic period (17.00%) and the non-pandemic period (18.60%) (*p* = 0.599), which indicated the stability of the occurrence of this type of injury regardless of external conditions.

Zygomatic arch injuries occurred in 4.12% of cases, with minimal differences between the pandemic period (4.05%) and the non-pandemic period (4.16%) (*p* = 0.945), indicating a similar distribution of the frequency of this type of injury in both periods.

Similarly, fractures of the orbit were recorded in 4.40% of cases, with slight differences between the pandemic period (4.45%) and the non-pandemic period (4.38%) (*p* = 0.962), which indicated no significant impact of the pandemic on the occurrence of these injuries.

Fractures of the nasal bone were identified in 5.97% of the total cases, with a frequency of 7.29% during the pandemic and 5.25% in the non-pandemic period, showing no significant statistical difference (*p* = 0.276).

Fractures of the frontal bone were reported in 1.42% of the total cases; only 0.40% occurred during the pandemic period compared to 1.97% during the non-pandemic period, suggesting a trend but without statistical significance (*p* = 0.178).

Multi-site head and facial fractures were identified in 1.42% of the entire sample, with a frequency of 0.81% during the pandemic and 1.75% outside of it, with no statistical difference (*p* = 0.507).

The severity of the maxillofacial injury was also assessed using the facial injury severity scale (FISS). The scale was proposed in 2006 by Bagheri et al. The final FISS score was the sum of all the individual scores. Based on the anatomical location (upper, middle, or lower facial level), the FISS classifies and assesses maxillofacial fractures [7]. However, the bone classification is not detailed enough and cannot be used to distinguish between displaced and comminuted fractures [8]. The use of this system to assess the severity of injuries is the current standard of care [7].

The data are shown in Table 2.

## 4. Discussion

Our study analysed the differences in the occurrence of various facial features and injuries in patients depending on the phase of the COVID-19 pandemic, considering before, during, and after its course. The impact of the pandemic’s social changes and pandemic-related restrictions on injury patterns was assessed. The results suggest that the COVID-19 pandemic may have changed trauma patterns in the facial region, leading to an increased risk of certain types of injuries, such as injuries to the mandibular condyle (especially the left side), Le Fort type II/III fractures, injuries to the maxillary alveolar process, or displacement of the upper facial mass. According to McManus, a professor of psychology at the University College London, around 90% of people are right-handed, which explains why the left side of the face is the most common site of injury [9]. At the same time, a reduction in the incidence of other injuries was observed, e.g., Le Fort III fractures or Le Fort type I/II fractures. This may suggest that the pandemic and its accompanying circumstances, such as changes in social behaviour, mobility, restrictions, and availability of healthcare, had an impact on the nature and distribution of craniofacial injuries. The results of the analysis on the effects of the COVID-19 pandemic on individual types of facial fractures prompt several additional reflections from a medical perspective. A significant increase in the risk of comminuted jaw fractures during the pandemic may have been related to changes in injury mechanisms. Restrictions in professional and social activities, as well as increased levels of stress and anxiety amongst the population, may have led to an increase in the frequency of high-energy injuries, such as falls from heights or traffic accidents, which are typical causes of comminuted fractures. The lack of a significant impact of the pandemic on most types of fractures analysed suggests that the overall epidemiological profile of craniofacial injuries remained relatively stable, despite significant changes in the functioning of society. This may have been due to the fact that craniofacial injuries are associated with risk factors such as traffic accidents, interpersonal violence, and sports injuries, the incidence of which may not have been drastically impacted by the pandemic itself. The observed trends in increased risk of maxillary alveolar process, coronoid process, and nasal bone fractures during the pandemic, although not statistically significant, may reflect the impact of pandemic-related factors on low-energy injuries. For example, an increased incidence of falls at home or injuries related to physical activity undertaken in confined conditions may have contributed to the increased risk of these types of fractures [6].

Based on these conclusions, comparisons were made with the data available from other articles on similar topics. A study conducted at the medical centre of Galilei, Nahariya, Israel showed that most fractures occurred in both the lower and middle parts of the face, which was consistent with our research [10]. However, the conclusion that there were no injuries to the upper part of the face during the lockdown was opposite to the results of our research. A conclusion extrapolated from data in the Terni province of Umbria, Italy indicated the most fractured anatomical site of the face was the orbital floor, then the zygoma and nasal bones [11]. However, in our study, these results were statistically insignificant. A study comparing oral and maxillofacial injuries during the first and third lockdowns of the COVID-19 pandemic in the United Kingdom showed an increase in orbital and soft tissue injuries [12]. Our study did not take into account soft tissue trauma; however, regarding injuries of the orbit, there was no significant impact of the pandemic on the occurrence of this type of injury. Our conclusion was therefore not consistent with the research results from the United Kingdom. An increase in the severity of oral and maxillofacial injuries, and subsequently general injuries, was found in a study conducted in the United States [13]. We did not take into account general injuries; however, regarding maxillofacial injuries, there was no significant impact of the pandemic on most of the analysed fractures, which was not in agreement with the conclusions from the study conducted in the United States. A study by Dawoud et al. aimed to investigate the impact of the lockdown during the SARS-CoV-2 pandemic on craniofacial injuries at a Level I trauma referral centre. The comparative study analysed data on patients admitted with craniofacial injuries during the lockdown period between March 15 and June 15, 2020, compared to the same period in 2019. The results showed that, despite the lockdown, there was no significant reduction in the volume of craniofacial injuries. This conclusion was consistent with our research. Patients during the lockdown were more likely to suffer from polytraumatic injuries [14]. We did not take polytrauma into account. With the introduction of e-scooters in Rome, Lazio, Italy, the most recorded fractures were those to the nasal bones, mandibular fractures, zygomaticomaxillary fractures, complex fractures, and maxilla [15]. The findings regarding injuries affecting the mandible or maxilla were consistent with our research. Research conducted in Nashville, Tennessee, aimed to investigate the impact of the 2020 lockdown on craniofacial injuries. This analysis showed that the lockdown was associated with a significant decrease in the number of patients with craniofacial injuries [16]. This was not in agreement with our study, where we found that there was no significant impact of the pandemic on most types of fractures analysed. The overall epidemiological profile of craniofacial injuries remained relatively stable. A study conducted in the Hospital of João XXIII in Santa Clara, Santarém, Brazil aimed to investigate the impact of the lockdown on maxillofacial surgery at a Level I Traumatology Hospital during the COVID-19 pandemic. The most common diagnoses during the years 2019 and 2020 were fractures of the orbit, maxilla, mandible, and zygomatic bone. Less frequently, the nasal bones were affected. A reduction of 77.77% was observed in major fractures involving the maxilla and mandible [17]. The above results were in agreement with ours in terms of an increased number of injuries to the maxilla and mandible. In Germany, research showed a decrease in the frequency of mandible fractures of different fracture locations. However, an increased frequency was observed in concomitant facial soft tissue injuries and traumatic tooth loss [18]. The conclusion regarding jawbone trauma was not in agreement with our research. However, the results for soft tissue or dental trauma cannot be compared because our study did not take into account such criteria. One study aimed to investigate the impact of the 2020 coronavirus lockdown on the presentation of oral and craniofacial injuries at a central hospital in London. A decrease in the number of bony trauma cases, dental trauma cases, and soft tissue trauma cases was identified [19]. We did not take into account dental or soft tissue trauma. Nevertheless, this information is undoubtedly important within the research conducted to analyse the impact of the COVID-19 lockdown on the epidemiology of craniofacial injuries. An increased incidence of fractures, especially Le Fort type fractures, was seen in a comparative study conducted in 13 major French public hospitals. The distribution of other types of fractures was similar, with a predominance of mandibular fractures [20]. The Le Fort fracture findings were partially consistent with our findings. In France, no division was specified as in our study; we found an increased risk of Le Fort II/III fractures and a reduction of Le Fort III fractures and Le Fort I/II fractures. Another study aimed to investigate changes in the epidemiology and aetiology of craniofacial injuries during the COVID-19 pandemic in Italy. The study showed a significant decrease in injuries affecting the nasal bones and an increase in those affecting the frontal bones [21]. However, in our study, there was an increase in the number of nasal bone injuries and a decrease in frontal bone injuries. A comparative study from the United Kingdom and Australia found that COVID-19-related social distancing measures influenced the epidemiology of facial injuries. During the 2020 study period, the number of facial trauma presentations decreased in both the UK and Australia compared to the previous year. The frequency of occurrence of mandibular fractures in Australia increased as mandibular alveolar fractures in the UK increased. The UK reported a significant decrease in the occurrence of soft tissue injuries [22]. The conclusions from the UK and Australia regarding jawbone injuries differed from ours. We cannot comment on soft tissue injuries because we did not consider this criterion in our study. A further study aimed to assess the impact of COVID-19 restrictions on the number of cases of craniofacial trauma by comparing data in India from the lockdown period with data from the previous year. A drastic decline in the total number of maxillary and mandibular fracture patients occurred during the lockdown, with a 73% reduction observed [23]. We did not reach similar conclusions in our study.

The results of our study analysing differences in the occurrence of various features and craniofacial injuries in patients depending on the phase of COVID-19 disease were similar to the results of other studies on the impact of the pandemic on craniofacial injuries. Despite several similar articles existing in the literature, there was no information about the precise specificity of fractures. In the available literature, we found retrospective studies comparing the causes of injuries during the COVID-19 period and the non-pandemic period. These studies considered injury under the influence of alcohol or not and recorded demographic information such as age, gender, and status, as well as whether the treatment was conservative or surgical [24]. However, many of these studies found a reduction in the number of injury cases during the lockdown period, which may have been related to the reduction in social activity and movement. In other words, there were some differences in the data presented; for example, some studies suggested an increase in injury cases during the pandemic, which may have been due to increased risks associated with physical activity at home or limited availability of medical services.

## 5. Conclusions

The COVID-19 pandemic had a significant impact on the location and frequency of facial skull injuries in the patients treated at the Department of Maxillofacial Surgery during the period from March 2019 to August 2023. The clinical implications of the pandemic period, in terms of oromaxillofacial trauma, included a significant increase in the risk of comminuted jaw fractures. The increased levels of stress and anxiety in the population may have led to an increase in the incidence of high-energy trauma. Moreover, the lack of a significant impact of the pandemic on most types of fractures analysed suggested that the overall epidemiological profile of craniofacial injuries remained relatively stable, despite significant changes in the functioning of society.

## Figures and Tables

**Table 1:** A compilation of the oromaxillofacial injury characteristics seen during the pandemic in the total sample and stratified by the examined period.

Characteristic	Total Sample ^a^	Examined Period		
		*p* ^b^		
		Pandemic Period *^a^*, *n*_1_ = 247	Non-Pandemic Period *^a^*, *n*_2_ = 457	
fracture of the angle of the mandible				0.623
left side	120.00 (61.54%)	39.00 (62.90%)	81.00 (60.90%)	
bilaterally	8.00 (4.10%)	1.00 (1.61%)	7.00 (5.26%)	
right side	67.00 (34.36%)	22.00 (35.48%)	45.00 (33.83%)	
	195	62	133	
fracture of the body of the mandible in the middle				0.907 *^c^*
no	634.0 (90.06%)	222.00 (89.88%)	412.00 (90.15%)	
yes	70.00 (9.94%)	25.00 (10.12%)	45.00 (9.85%)	
	704	247	457	
mandibular body fracture				0.808 *^c^*
left side	102.00 (42.86%)	35.00 (40.70%)	67.00 (44.08%)	
bilaterally	12.00 (5.04%)	5.00 (5.81%)	7.00 (4.61%)	
right side	124.00 (52.10%)	46.00 (53.49%)	78.00 (51.32%)	
	238	86	152	
fracture of the mandibular ramus	43.00 (6.11%)	17.00 (6.88%)	26.00 (5.69%)	0.528 *^c^*
fracture of the condylar process of the mandible				
left side	61.00 (37.20%)	38.00 (55.88%)	23.00 (23.96%)	<0.001 *^d^*
bilaterally	61.00 (37.20%)	5.00 (7.35%)	56.00 (58.33%)	<0.001 *^d^*
right side	42.00 (25.61%)	25.00 (36.76%)	17.00 (17.71%)	<0.001 *^d^*
	164	68	96	
fracture of the head of the mandible	17.00 (2.41%)	7.00 (2.83%)	10.00 (2.19%)	0.594 *^c^*
fracture of the coronoid process of the mandible	17.00 (2.41%)	8.00 (3.24%)	9.00 (1.97%)	0.295 *^c^*
fracture of the alveolar part of the mandible	8.00 (1.14%)	3.00 (1.21%)	5.00 (1.09%)	1.000
jaw fractures Le Fort				
Le Fort type I	16.00 (23.19%)	6.00 (27.27%)	10.00 (21.28%)	0.838 *^d^*
Le Fort type II	33.00 (47.83%)	15.00 (68.18%)	18.00 (38.30%)	0.201 *^d^*
Le Fort type III	20.00 (28.99%)	1.00 (4.55%)	19.00 (40.43%)	0.004 *^d^*
	69	22	47	
complicated jaw fractures Le Fort				<0.001 *^c^*
Le Fort type I/II	36.00 (59.02%)	1.00 (6.25%)	35.00 (77.78%)	
Le Fort type II/III	25.00 (40.98%)	15.00 (93.75%)	10.00 (22.22%)	
	61	16	45	
fracture of the maxillary alveolar process	24.00 (3.41%)	13.00 (5.26%)	11.00 (2.41%)	0.046 *^c^*
fracture in the maxillary sinus	29.00 (4.12%)	11.00 (4.45%)	18.00 (3.94%)	0.743 *^c^*
displacement of the upper facial mass	17.00 (2.41%)	11.00 (4.45%)	6.00 (1.31%)	0.010 *^c^*
zygomaticomaxillary—orbital fracture	127.00 (18.04%)	42.00 (17%)	85.00 (18.60%)	0.599 *^c^*
fracture of the zygomatic arch	29.00 (4.12%)	10.00 (4.05%)	19.00 (4.16%)	0.945 *^c^*
fracture in the orbit	31.00 (4.40%)	11.00 (4.45%)	20.00 (4.38%)	0.962 *^c^*
fracture of the nasal bone	42.00 (5.97%)	18.00 (7.29%)	24.00 (5.25%)	0.276 *^c^*
fracture in the frontal bone	10.00 (1.42%)	1.00 (0.40%)	9.00 (1.97%)	0.178
multi-site head and facial fractures	10.00 (1.42%)	2.00 (0.81%)	8.00 (1.75%)	0.507

*^a^ n* (%), *^b^* Fisher’s exact test, *^c^* Pearson’s chi-squared test, *^d^* proportion test.

**Table 2:** FISS: facial injury severity scale.

Fracture Type	Score
fracture of the angle of the mandible (left side, bilaterally, right side)	2
fracture of the body of the mandible in the middle	2
mandibular body fracture (left side, bilaterally, right side)	2
fracture of the mandibular ramus	2
fracture of the condylar process of the mandible (left side, bilaterally, right side)	1
fracture of the head of the mandible	1
fracture of the coronoid process of the mandible	1
fracture of the alveolar part of the mandible	1
Le Fort type I	2
Le Fort type II	4
Le Fort type III	6
Le Fort type I/II	2/4
Le Fort type II/III	4/6
fracture of the maxillary alveolar process	1
fracture in the maxillary sinus	1
displacement of the upper facial mass	5
zygomaticomaxillary orbital fracture	1
fracture of the zygomatic arch	1
fracture in the orbit	1
fracture of the nasal bone	1
fracture in the frontal bone	1
multi-site head and facial fractures	5

## Data Availability

The data on which this study is based will be made available upon request.
